# An Explainable Hybrid CNN–Transformer Architecture for Visual Malware Classification

**DOI:** 10.3390/s25154581

**Published:** 2025-07-24

**Authors:** Mohammed Alshomrani, Aiiad Albeshri, Abdulaziz A. Alsulami, Badraddin Alturki

**Affiliations:** 1 Department of Computer Science, Faculty of Computing and Information Technology, King Abdulaziz University, Jeddah 21589, Saudi Arabia; aaalbeshri@kau.edu.sa; 2 Department of Information Systems, Faculty of Computing and Information Technology, King Abdulaziz University, Jeddah 21589, Saudi Arabia; aaalsulami10@kau.edu.sa; 3 Department of Information Technology, Faculty of Computing and Information Technology, King Abdulaziz University, Jeddah 21589, Saudi Arabia; baalturki@kau.edu.sa

**Keywords:** malware classification, deep learning, convnext, vision transformer, explainable AI, Grad-CAM, cybersecurity

## Abstract

Malware continues to develop, posing significant challenges for traditional signature-based detection systems. Visual malware classification, which transforms malware binaries into grayscale images, has emerged as a promising alternative for recognizing patterns in malicious code. This study presents a hybrid deep learning architecture that combines the local feature extraction capabilities of ConvNeXt-Tiny (a CNN-based model) with the global context modeling of the Swin Transformer. The proposed model is evaluated using three benchmark datasets—Malimg, MaleVis, VirusMNIST—encompassing 61 malware classes. Experimental results show that the hybrid model achieved a validation accuracy of 94.04%, outperforming both the ConvNeXt-Tiny-only model (92.45%) and the Swin Transformer-only model (90.44%). Additionally, we extended our validation dataset to two more datasets—Maldeb and Dumpware-10—to strengthen the empirical foundation of our work. The proposed hybrid model achieved competitive accuracy on both, with 98% on Maldeb and 97% on Dumpware-10. To enhance model interpretability, we employed Gradient-weighted Class Activation Mapping (Grad-CAM), which visualizes the learned representations and reveals the complementary nature of CNN and Transformer modules. The hybrid architecture, combined with explainable AI, offers an effective and interpretable approach for malware classification, facilitating better understanding and trust in automated detection systems. In addition, a real-time deployment scenario is demonstrated to validate the model’s practical applicability in dynamic environments.

## 1. Introduction

Cybersecurity has seen an alarming rise in sophistication over the past decade, and malware remains a major challenge for digital systems, as attackers frequently find unique methods to slip past security measures. Recently, the Kaspersky Security Network reported that ransomware attacks increased globally from 2023 to 2024, reaching 0.44% of users, up by 0.02 percentage points. The hardest-hit areas were the Middle East, APAC, and Africa, where rapid digital transition and inconsistent cybersecurity techniques have made systems more vulnerable [1]. Conventional detection techniques, mainly those that rely on static signature matching, struggle to keep up with the current malware development. Adversaries now operate advanced techniques [2], including polymorphism, encryption, and code packing, which can bypass conventional antivirus solutions [3]. This has motivated the security research community to explore alternative detection approaches that are more robust, adaptive, and intelligent. One approach that has recently gained increasing interest involves turning malware binaries into visual images. In this approach, the raw binary files are represented as a grayscale image, and this transformation makes it possible to apply advanced image recognition techniques, many of which have shown strong results in areas such as medical imaging [4], autonomous vehicles [5], and visual object tracking [6]. When malware is approached as an image classification task, researchers can detect subtle visual cues, structural patterns, and spatial features that are often missed during standard binary-level analysis.

There are several benefits to representing malware as images. Foremost, image-based models can detect structural similarities between different malware instances. This conversion facilitates the benefit of well-established convolutional neural network (CNN) architectures to extract hierarchical features from visual data. Eventually, this method provides an intuitive visual medium for cybersecurity experts to study the image-like artifacts to better understand how malware families behave.

Although CNNs have been widely used in malware image classification tasks, they are not without limitations. CNNs are primarily designed to capture local patterns through small convolutional filters. Although this works well for detecting edges, textures, and other low-level features, they are less effective at modeling long-range dependencies or global relationships within an image. In contrast, Vision Transformers (ViTs) have recently emerged as an alternative deep learning paradigm that uses self-attention mechanisms to process images. These models can capture dependencies across distant regions of the image and are particularly good at understanding the overall context of visual scenes. However, each architecture has its trade-offs. CNNs are more efficient and easier to train with limited data but may overlook global patterns. Vision Transformers require more data and computational resources but offer stronger performance on tasks requiring holistic understanding. This prompts a critical research question: “Is it possible to integrate the strengths of both architectures to enhance malware classification performance”?

In this paper, we explore a hybrid approach that combines a CNN-based backbone (ConvNeXt-Tiny) and a Transformer-based backbone (Swin Transformer) into a unified model for malware classification. The ConvNeXt component captures fine-grained, low-level features, while the Swin Transformer contributes a global perspective by modeling relationships across the entire image. This hybrid design aims to improve the classification accuracy while maintaining computational efficiency. In contrast, in deep learning applications where security is a primary concern, model interpretability becomes just as crucial as predictive accuracy. A system designed to detect malware must not only perform well but also provide insight into its decision-making process. Without transparency, even accurate models may struggle to accumulate the trust of cybersecurity analysts. To address this, we incorporate Grad-CAM, an explainable AI method to highlight the image regions most influential in the predictions. By visualizing these focus areas, Grad-CAM allows us to assess whether the model is attending to meaningful features associated with malicious activity, thereby supporting both validation and operational trust.

Despite growing interest in visual malware classification, most prior studies have focused on CNNs or Transformers in isolation. Moreover, very few works have attempted to make these models explainable. Our research fills this gap by developing a novel hybrid model that fuses CNN and Transformer backbones, and we apply Grad-CAM to interpret and analyze the model decision-making process. There are alternative or enhanced mechanisms—such as token mixers (e.g., MLP-Mixer) or graph-based neural networks—that could offer a promising direction for malware classification. However, our current proposed model was guided by a balance between model expressiveness and architectural stability for image-based malware patterns. Moreover, the Swin Transformer was selected due to its hierarchical structure and window-based attention, which naturally align with the texture-block nature of malware images. We trained and tested our approach using three publicly available datasets—Malimg, MaleVis, and VirusMNIST—covering 61 different malware families. Our experiments show that the hybrid model achieved a higher validation accuracy (94.04%) compared to models using ConvNeXt-Tiny alone (92.45%) or Swin Transformer alone (90.44%). We also validated our proposed model using two extended datasets, Maldeb and Dumpware-10. In addition to accuracy improvements, the Grad-CAM visualizations offer practical insights into how the model processes malware images and where it focuses its attention.

The central challenge addressed in this study is how to design an effective, interpretable deep learning model for malware image classification that can handle the wide variability and structural complexity of real-world malware. Traditional models either fail to capture global context or are difficult to interpret. We aim to resolve these shortcomings by integrating a hybrid CNN–Transformer architecture with an explainable AI framework. To summarize, this paper makes the following contributions:

- We propose a novel architecture that combines ConvNeXt-Tiny (CNN) with Swin Transformer (ViT) for malware image classification, enabling the extraction of both local and global features.
- We systematically apply Grad-CAM to hybrid visual malware models, enabling analysts to visualize and interpret the internal decision-making process.
- We conduct extensive experiments on three benchmark malware image datasets (Malimg, MaleVis, and VirusMNIST), demonstrating improved classification performance and model reliability. Also, we extend our validation to two more datasets—Maldeb and Dumpware-10—to show the model’s generalization ability and practical applicability across varied threat landscapes.
- This study bridge the gap between deep learning models and the interpretability required for cybersecurity, providing a practical solution for real-world deployment.

The remainder of the paper is structured as follows. Section 2 reviews related research on Machine Learning (ML) and Deep Learning (DL), with the DL section further divided into subsections covering CNN-based models, Vision Transformer-based models, and CNN+Transformer hybrid models for visual malware classification and explainable AI (XAI) in cybersecurity. Section 3 describes the research methodology and design. Section 4 discusses the results of the research. Section 5 presents the real-world deployment scenario. Section 6 concludes and discusses future work.

## 2. Literature Review

### 2.1. Machine Learning Models for Malware Classification

There are few research works that have been done on malware classification using classical machine learning. Luo and Lo [7] proposed a malware classification approach based on dividing malware images into 3 × 3 grids and extracting Local Binary Pattern (LBP) features. These features were used as input for CNN, SVM, and k-NN and tested on 32 malware families. Among the models, the CNN model achieved the highest accuracy of 93.17%, outperforming SVM and k-NN, which obtained 87.88% and 85.93%, respectively. While these results demonstrate the potential of combining LBP features with ML models, the method has certain limitations. The LBP is sensitive to image resolution and may struggle to capture high-level structural patterns as the image size increases. Moreover, relying on handcrafted features restricts the model’s adaptability to evolving malware variants. Son et al. [8] introduced an efficient image-based malware classification approach that avoids conventional square image inputs by resizing malware images to vertically compressed formats (e.g., 8 × 64, 16 × 64, 32 × 64) based on the observation that malware feature patterns are primarily vertically distributed. Unlike prior works relying on feature extractors like GIST or LBP, the authors directly input these reshaped images into k-NN, SVM, and CNN classifiers. They achieved the highest 98.11% accuracy on the MalImg dataset using KNN and 8 × 64 image dimensions for 0.25 s of training time. Experimental results on the Malimg and Malheur datasets showed that reduced image dimensions significantly lowered training time—up to 8×—without compromising classification accuracy. In a related study [9], the authors evaluated the impact of image resolution on classification performance using the k-NN algorithm on the Malimg dataset. They tested images resized to 128 × 128, 64 × 64, 32 × 32, and 16 × 16 pixels, with resulting accuracies of 52.71%, 83.33%, 97.9%, and 97.62%, respectively. These findings suggest that as the image resolution increases, the performance of k-NN declines—likely due to the algorithm’s reliance on Euclidean distance in high-dimensional spaces, which can degrade its effectiveness.

While some studies have applied classical ML models such as k-NN to malware image datasets (e.g., Malimg), their performance has been found to degrade with increasing image resolution—highlighting a limitation in handling high-dimensional visual data. This motivates the use of deep learning architectures that can better capture local and global patterns in image-based malware representations. Since our study focuses on end-to-end vision-based learning using 224 × 224 malware images, deep architectures such as CNNs and Transformers are more suitable.

### 2.2. Deep Learning Models for Malware Classification

Most research emphasizes the importance of behavior analysis techniques compared to traditional signature-based methods. This study divided the litearture review on deep learning models used for malware classification into three sections, namely, CNN-based models, Transformer-based models, and CNN+Transformer-based hybrid models.

#### 2.2.1. CNN-Based Models

The concept of visualizing malware as images was presented by Nataraj et al. [10], who introduced the Malimg dataset by converting malware binaries into grayscale images. Their work demonstrated that image-based malware classification could achieve high accuracy using simple texture analysis techniques. Subsequently, numerous studies have explored this approach, with researchers developing various visualization techniques for malware binaries. Awan et al. [11] proposed a deep learning framework, SACNN, which combines spatial attention with the CNN for image-based classification using the Malimg dataset. The model achieved high performance across multiple metrics, with the precision, recall, and F1-scores all exceeding 97%, both with and without class balancing. While the model is relatively simple compared to more complex architectures, its strong accuracy demonstrates that lightweight designs can still be effective for malware detection, though broader generalization beyond Malimg remains to be explored. Singh et al. [12] developed a hybrid malware classification model that combines Gated Recurrent Units (GRUs) for sequential feature extraction with a CNN-based feature refiner, followed by classification using a Cost-sensitive Bootstrapped Weighted Random Forest (CSBW-RF). The approach achieved 99.58% accuracy on the Malimg dataset and demonstrated strong generalizability on the Microsoft Big 2015 dataset, outperforming several existing models. While the method shows high robustness and adaptability across datasets, its complexity and reliance on multiple processing stages may pose challenges for real-time deployment in constrained environments. Kumar et al. [13] introduced transfer learning and ensemble learning for malware classification, achieving an accuracy of 99.36% on the Malimg test dataset and 92.11% on a real-world malware dataset. Kalash et al. [14] proposed a CNN-based malware classification model by converting binary images to grayscale. They evaluated the proposed model on the the Malimg and Microsoft malware datasets and achieved 98.52% and 99.97% accuracy results, respectively. Ravi and Alazab [15] proposed an attention-based CNN method and achieved a 99% accuracy result on the MalImg dataset. Panda et al. [16] introduced a stacked ensemble model (SE-AGM), combining an autoencoder, GRU, and MLP, trained on 25 extracted features from the MalImg dataset to classify malware families efficiently. Leveraging CNN-based transfer learning and data augmentation, their model achieved a high accuracy of 99.43%, outperforming several baseline approaches. Despite its strong performance, the model’s reliance on a limited feature set and evaluation on a single dataset may restrict its adaptability to broader malware environments. Alam et al. [17] proposed an efficient layered feature extractor combined with spatial-CNN, which attained 99.87% on Malimg, 99.81% on BIG2015, and 99.22% on MaleVis, to design a streamlined architecture for malware classification. Guan et al. [18] presented a hybrid of ResNet50 and VGG16 using knowledge distillation, achieving a 99.50% accuracy result on Malimg and 97.52% accuracy result on BIG2015, to compress deep models while retaining high accuracy through fused layer optimization. Abdulazeez et al. [19] benchmarked DenseNet201 combined with KNN on malware data, obtaining a 96% accuracy result on Malimg, to evaluate the suitability of pretrained models for malware detection tasks. Alnajim et al. [20] explored CNN and DNN models, reporting a 98.14% accuracy result on Malimg and 98.95% accuracy result on BIG2015, to broaden deep learning-based approaches for malware analysis.

The MaleVis dataset [21] contains malware samples generated using a diverse approach that emphasizes the structural and behavioral characteristics of malware samples. Al-Khater and Al-Madeed [22] addressed the challenge of detecting new malware by applying the fast and adaptive bidirectional empirical mode decomposition technique to improve the quality of the dataset and overcome class imbalance. Their study evaluated two 3D deep learning architectures, VGG-16 and ResNet-18, and their performance outcomes on the Malimg and MaleVis datasets, achieving up to 99.64% precision with ResNet-18. While the results are promising, the approach relies on computationally intensive 3D models and preprocessing steps, which may affect their scalability and real-time applicability in practical cybersecurity settings. Atitallah et al. [23] introduced a vision-based IoT malware detection framework using deep transfer learning and ensemble methods to improve classification performance. Their approach combines ResNet18, MobileNetV2, and DenseNet161 through a random forest voting strategy, and it was evaluated on the MaleVis dataset. The model achieved a 98.68% accuracy result. However, the method relies on RGB image transformation and ensemble complexity, which may limit its real-time deployment in resource-constrained IoT environments.

Noever and Miller introduced the VirusMNIST dataset [24], which includes more than 50,000 virus examples from nine malware families and benign files. They implemented a MobileNetV2 CNN model, achieving an accuracy of 80% in classifying malware samples. Their study highlighted the challenges of distinguishing between malware families with similar visual patterns. Habibi et al. [25] addressed the limitations of conventional malware detection by employing CNN and transfer learning models—MobileNetV2 and ResNet50—for robust classification of malware, including obfuscated variants. The proposed models trained on VirusMNIST and achieved 99% accuracy results in general classification and 100% accuracy results in obfuscated malware detection on the Malimg dataset. While the results demonstrate high effectiveness, the study primarily evaluated static visual patterns and may benefit from additional analysis on dynamic behavior or unseen zero-day threats. Zou et al. [26] introduced FACILE, a capsule network optimized for malware classification that integrates dynamic convolution and balanced routing to reduce training complexity and improve feature representation. Tested on the VIRUS-MNIST, MalImg, and BIG2015 datasets, FACILE reduced error rates to 8.087%, 1.149%, and 2.797%, respectively. Dutta et al. [27] proposed KOL-4-GEN, a suite of four deep learning models based on Kolmogorov–Arnold Networks with trainable activation functions, integrated with a GAN to mitigate data imbalance during malware image classification. Evaluated on the Malimg, Malevis, and Virus-MNIST datasets, the models achieved validation accuracies of approximately 99.36%, 95.44%, and 92.12%, respectively.

Other research works have also been proposed for malware classification using a deep learning model. For example, Yang et al. [28] proposed a malware detection framework that combines binary and opcode features using a stacked convolutional network and a triangular attention mechanism. Their model employs cross-attention to align and fuse feature representations, achieving 99.54% accuracy on the Kaggle Malware Classification dataset and 95.44% on a real-world dataset. A visualized attention module further enhances interpretability by highlighting relevant opcode patterns. However, the approach is limited to sequential features and does not explore visual or Transformer-based architectures to capture spatial or global dependencies. Cui et al. [29] proposed a grayscale-based visualization method that converted malicious code. They implemented the CNN to extract visual features and addressed the imbalanced dataset using the BAT algorithm. Abdullah et al. [30] proposed a hybrid static classifier that combines CNN and BILSTM to detect malware in IoT environments using 1D image representations of Byte and Assembly files. The model achieved average accuracies of 99. 91% and 99. 83% in the Microsoft Malware Classification and IoT Malware datasets, respectively. Although the method benefits from automatic feature extraction and strong accuracy, its reliance on 1D image conversion and dual-stage architecture may limit its adaptability to other data formats. Brosolo et al. [31] evaluated visual malware analysis techniques and discussed key challenges such as the lack of interpretability of deep learning models. Karat et al. [32] proposed a CNN-LSTM algorithm for zero-day malware detection. They used two API call sequence datasets to validate the proposed methods and achieved a 96% validation accuracy. There are also several research works that have explored alternative datasets beyond the commonly used benchmarks. For example, Chaganti et al. [33] applied CNNs combined with feature fusion techniques, achieving a 97% accuracy result on a malware dataset, with the goal of enhancing classification performance through multi-feature integration. Ahmed et al. [34] utilized InceptionV3 to achieve a 98.76% accuracy result on BIG15, aiming to compare machine learning and transfer learning approaches for malware detection. Ismail et al. [35] employed contrastive learning and data augmentation, with pretraining performed on the unlabeled ImageNet dataset, followed by fine-tuning on unlabeled malware samples. The system was evaluated through two downstream tasks: malware family classification and malware-versus-benign detection. Their experimental results reported a 98.4% accuracy result on the Malimg dataset and 96.2% on the Maldeb dataset, surpassing the performance of existing self-supervised approaches. Puneeth et al. [36] employed a CNN-based approach for malware classification and evaluated it on the Binary, Malimg, and Dumpware-10 datasets, achieving accuracies of 99.15%, 99.26%, and 98.19%, respectively.

#### 2.2.2. Transformer-Based Models

Initially developed for natural language processing, Transformers have recently been repurposed for applications in computer vision. Dosovitskiy et al. [37] introduced the Vision Transformer (ViT), demonstrating its competitive performance in image classification by conceptualizing images as sequences of patches. Following this work, numerous variants have been proposed to overcome the limitations inherent in the original ViT architecture [38]. Liu et al. [39] introduced the Swin Transformer, which implements shifted windows to improve computational efficiency and effectively capture local dependencies. This architecture has demonstrated strong performance across various computer vision tasks, including image classification, object detection, and semantic segmentation. The transfer learning-based Butterfly Vision Transformer (B-ViT) is a widely used method for malware classification. For example, Belal et al. [40] implemented four types of B-ViTs, including B-VIT/B16, B-VIT/B32, B-VIT/L16, and B-VIT/L32. They evaluated the proposed methods using the Malimg, Microsoft BIG, and PE imports datasets and achieved accuracy results of 99.32%, 99.49%, and 99.99%, respectively. Ashawa et al. [41] implemented ResNet-152 and the Vision Transformer architecture and achieved a 99.62% accuracy result using 10-fold cross-validation for malware classification.

Wang et al. [42] employed a self-supervised Swin Transformer that reached 97.85% on BIG2015 and 98.28% on Malimg in terms of accuracy, focusing on developing a lightweight yet effective framework for malware image classification. Zhao et al. [43] integrated a Swin Transformer with deformable attention, achieving a 99.35% accuracy result on Malimg, to enhance classification performance through improved attention mechanisms. Ahmed et al. [44] proposed Gaussian Discriminant Analysis (GDA) and Segmentation-based Fractal Texture Analysis (SFTA) for feature extraction and Naive Bayes (NB) for classification, achieving a 98% accuracy result on MaleVis, aiming to assess the effectiveness of Vision Transformer models for binary and multi-class malware classification tasks. Ashwini et al. [45] introduced a dual Vision Transformer with split attention, which attained a 99.99% accuracy result on a ransomware dataset, focusing on improving ransomware detection using attention-enhanced Transformer models.

#### 2.2.3. CNN+Transformer-Based Hybrid Models

Rahman et al. [46] proposed a hybrid data augmentation technique combining the Deep Convolutional Generative Adversarial Network and conventional image transformation methods. The classifier was used on the MalImg dataset and trained with the proposed augmentation technique, and it achieved a 99.94% accuracy result for six-class classification and a 99.79% accuracy result for 25-class classification. Rahman et al. [47] compared CNN, ViT, CCT, and EANet models with LIME explainability, where the CNN model achieved a 99.44% accuracy result on Malimg, to analyze the effectiveness of CNN versus Transformer architectures on malware images.

While numerous hybrid architectures exist in the broader deep learning literature, our proposed model is specifically tailored for image-based malware classification, a domain with unique visual and structural characteristics. Unlike existing hybrids that typically employ static fusion (e.g., simple concatenation or parallel outputs), our approach introduces a dynamic feature fusion mechanism using a learned gating unit, which adaptively balances the contributions from the ConvNeXt and Swin Transformer backbones. Additionally, our architecture incorporates auxiliary supervision and knowledge distillation, allowing the model to benefit from intermediate representations during training and enabling efficient transfer to lighter deployment variants.

### 2.3. Explainable AI in Cybersecurity

Although deep learning models have achieved impressive performance in various cybersecurity tasks, their black-box nature raises concerns about trustworthiness and interpretability. Explainable AI (XAI) techniques aim to address these concerns by providing insight into model decisions. Several studies have applied XAI techniques, such as LEMNA (Local Explanation Method using Nonlinear Approximation) [48], LIME (Local Interpretable Model-Agnostic Explanations) [49], and SHAP (SHapley Additive exPlanations) [49,50], to cybersecurity tasks.

Ullah et al. [51] proposed an explainable Android malware detection system that combines visual and textual features using transfer learning, malware image conversion, and the BERT model for semantic representation. The system integrates handcrafted feature extractors (FAST and BRIEF), applies SMOTE for class balancing, and employs CNNs and ensemble learning for classification, achieving strong performance on the CICMalDroid 2020 and CIC-InvesAndMal2019 datasets. While the approach effectively blends multi-modal features and enhances interpretability, its reliance on multiple complex processing stages may present challenges for deployment in low-resource mobile environments. Guo et al. [48] used LEMNA to explain network intrusion detection models. Prity et al. [49] implemented explainable AI methods for malware detection, such as SHAP and LIME. They validated the explanation using the chi-squared test. Table 1 summarizes the related malware classification studies by listing proposed methods, datasets and results, research objectives, and research limitations.

#### Research Gaps

Although substantial progress has been made in using deep learning and Transformer-based models for classifying malware images, studies exploring a combined or hybrid approach remain limited. Models based purely on CNNs tend to be strong in identifying localized features, while Transformers are better at capturing broader, contextual relationships. Bringing these two together could create a more balanced and effective model, but this potential remains largely untapped in the current literature. Another issue that stands out is that many works rely on just one dataset for evaluation. While that is often done for consistency or due to dataset constraints, it makes it hard to know whether a model will perform well across different scenarios. Using multiple datasets such as Malimg, MaleVis, VirusMNIST, Maldeb, and Dumpware-10 provides a broader basis for validating a model’s reliability and generalizability across different malware types. Interpretability is another area where most existing models fall short. Despite the push for AI in cybersecurity, we still do not see many models that clearly show why they made a particular decision. This lack of transparency can be problematic, especially in a domain where trust is critical, and the consequences of mistakes are high.

To address these gaps, our research proposes a hybrid malware classification model that brings together ConvNeXt-Tiny, a CNN backbone, with the Swin Transformer, which adds the ability to model global relationships in the data. We evaluated this model using all five datasets—Malimg, MaleVis, VirusMNIST, Maldeb, and Dumpware-10—to ensure broader applicability. To address the issue of interpretability, we also used Grad-CAM to visually map out which parts of an image influenced the model’s decision, providing some much-needed insight into its inner workings.

## 3. Methodology

**Figure 1:**
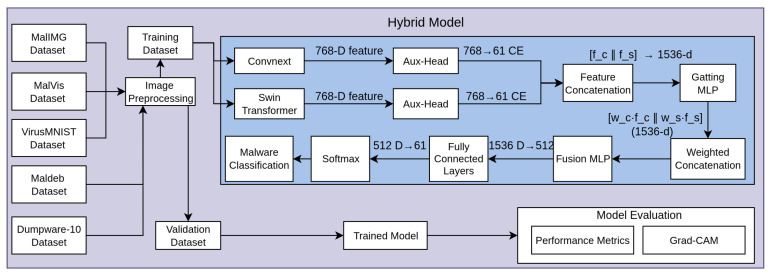
Proposed hybrid malware classification framework.

Figure 1 illustrates the general architecture of the proposed hybrid malware classification framework. The process begins with three publicly available datasets, MalIMG, MaleVis, VirusMNIST (see Section 3.1), which are first subjected to a unified image preprocessing step (see Section 3.2). The processed images are then split into training and validation sets. The proposed hybrid model contains two deep learning backbones (see Section 3.3), which are employed in parallel for feature extraction—ConvNeXt and Swin Transformer—each generating a 768-dimensional feature vector. These features are passed through auxiliary classification heads using a 768 → 61 fully connected layer trained with cross-entropy loss to aid convergence. The intermediate features from both backbones are concatenated, resulting in a 1536-dimensional joint representation. A gating MLP takes this concatenated vector and outputs softmax-normalized weights, which perform a weighted concatenation of the original features. The resulting 1536-dimensional fused vector is refined through a Fusion MLP (1536 → 512) followed by a final fully connected layer (512 → 61), which maps to the class space. The final classification is obtained using softmax. Model evaluation is conducted on the trained model using both standard performance metrics and interpretability analysis via Grad-CAM to visualize class-discriminative regions. Moreover, the Maldeb and Dumpware-10 datasets are used to validate our proposed model.

The proposed Algorithm 1 employs a hybrid deep learning framework to classify malware images by leveraging both ConvNeXt and Swin Transformer models. Initially, input images from datasets such as Malimg, MaleVis, and VirusMNIST undergo preprocessing steps like resizing and normalization. These processed images are then passed through ConvNeXt and Swin Transformer backbones, which extract 768-dimensional feature vectors independently. Each of these features is also processed through auxiliary classification heads that output 61-class logits, guiding the model via cross-entropy loss to enhance learning through additional supervision. The core features from both models are concatenated to form a 1536-dimensional vector, which is passed through a gating MLP to generate attention-based weights. These weights are used to construct a weighted fusion of the original features, again forming a 1536-dimensional representation. This fused vector is then processed through a fusion MLP and fully connected layers, reducing its dimensionality and producing final class predictions through softmax activation. In parallel, a knowledge distillation mechanism is integrated by comparing the student model’s predictions with those from a pretrained teacher using Kullback–Leibler divergence. The training objective combines the main classification loss, auxiliary loss, and distillation loss, enabling the model to benefit from both architectural diversity and prior knowledge. The model is trained iteratively and validated using a separate validation set to monitor performance and apply early stopping when necessary. Additionally, the proposed model was further validated by two datasets: Maldeb and Dumpware-10.**Algorithm 1:** Hybrid malware classification with adaptive fusion and knowledge distillation
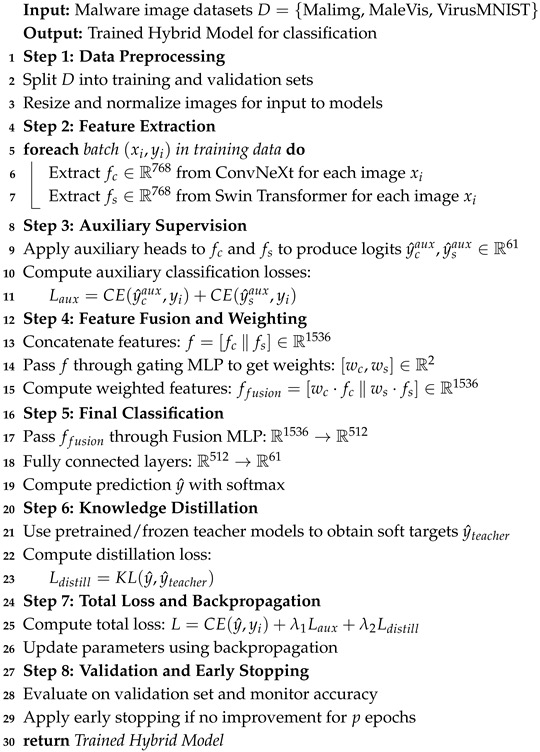


### 3.1. Datasets

In this study, we used a total of five datasets: Three benchmark datasets (Malimg, MaleVis, and VirusMNIST) were used for training and testing the model, while two additional datasets (Maldeb and Dumpware-10) were used for external validation of the proposed model.

Malimg: The Malimg [10] dataset contains 9339 malware grayscale images from 25 families. Each malware binary is interpreted as a vector of 8-bit unsigned integers and transformed into a 2D array, which is visualized as a grayscale image.

MaleVis: MaleVis [21] contains malware samples generated using a diverse approach that emphasizes the structural and behavioral characteristics of malware samples. The dataset has 26 classes, including 25 distinct forms of malware and benign samples, and it was preprocessed into 300 × 300 pixel images. It has a total of 9100 training and 5126 validation RGB images. All training classes consistently distributed 350 image samples; however, the validation set differs per class.

VirusMNIST: This dataset [24] contains 50,000 virus samples, including 9 malware families and a benign class. The nine malware families are generated from an unsupervised K-means clustering algorithm.

Maldeb: Maldeb [52] dataset comprises only two classes: benign (10,427 samples) and malicious (10,427 samples). Malware samples were collected from public repositories, including TekDefense, Malware-Repo, Malware Database, MalwareBazaar, and TheZoo. In contrast, benign samples were collected from legitimate system applications on Microsoft Windows 10 and 11, as well as from trusted open-source software platforms such as SourceForge, PortableFreeware, CNET, and FileForum. To ensure dataset integrity, all samples were verified using VirusTotal malware scanning services. For preprocessing, malware binaries were converted into grayscale images based on the methodology introduced by Nataraj [10].

Dumpware-10: This dataset [53] consists of 4294 grayscale images, including 3686 malware samples and 608 benign samples. Each image was resized into 224 × 224 pixels with a single channel. The dataset encompasses ten malware families and one benign class, ensuring class diversity for evaluating the classification model.

Table 2 categorizes the malware families from the Malimg, MaleVis and Dumpware-10 datasets based on their associated threat types [10]. For example, Adialer.C and Dialplatform.B belong to the Dialer threat category, while malware like Agent.FYI fall under the Backdoor threat category. Malware such as Allaple.A, Autorun.K, Autorun.PU, VB.AT, Yuner.A, and Fasong are categorized as Worms. Meanwhile, C2LOP.P, C2LOP.gen!g, Malex.gen!J, Skintrim.N, Androm, Expiro-H, Dinwod!rfn, Injector, Regrun.A, Snarasite.D!tr, Stantinko, VBKrypt, and Vilsel fall under the Trojan threat category.

Microsoft (microsoft.com) has identified a range of technical behaviors exhibited by various malware families, each demonstrating distinct malicious capabilities. The Allaple.A family, classified as a worm, is noted for its polymorphic nature, enabling it to propagate across local area networks (LANs) while launching denial-of-service (DoS) attacks. The Alueron.gen!J trojan is designed to compromise network security by altering DNS configurations on network routers, thereby redirecting traffic or facilitating further attacks. The C2LOP.P and C2LOP.gen!g trojans manipulate web browser settings, often adding unwanted bookmarks and altering configurations to serve the attacker’s objectives. Similarly, the Dontovo.A malware, categorized as a trojan downloader (TDownloader), facilitates the unauthorized downloading and execution of arbitrary files, paving the way for secondary infections. FakeRean pretends to scan PC malware. The Instantaccess dialer not only grants remote access to compromised systems but also modifies system registry entries to secure persistence or further exploitation. The Lolyda variants (Lolyda.AA1, Lolyda.AA2, and Lolyda.AA3) function as polymorphic droppers, delivering and installing additional malicious payloads. Lolyda.AT focuses on harvesting user credentials and transmitting the stolen information to remote servers controlled by the attacker. The Rbot!gen backdoor operates as an IRC-based bot that allows remote command and control, spreading laterally through networks by exploiting software vulnerabilities. Downloader families such as Swizzor.gen!E and Wintrim.BX primarily serve to retrieve and install additional malware components. Swizzor.gen!E functions as a generic downloader for diverse threats, whereas Wintrim.BX specifically installs further elements of the Wintrim malware suite. Adware and potentially unwanted applications (PUAs) present additional threats. Adposhel introduces unwanted files and registry modifications during installation, often to support intrusive advertising. Amonetize and Elex, both classified as PUAs, alter browser settings and shortcuts, leading to unwanted redirects or advertisements. BrowseFox not only modifies browser settings but also installs unsolicited browser extensions, further compromising user privacy and browser integrity.

The VirusMNIST dataset has been excluded from this table, as its classes were derived through unsupervised clustering rather than predefined malware families.

### 3.2. Image Preprocessing and Augmentation

This study resized all images to 224 × 224 pixels and converted the RGB images to grayscale images to keep consistency across datasets and compatibility with the pretrained models. We also implemented the following data augmentation techniques to balance the dataset images: random horizontal flipping, random rotation up to 10 degrees, color jitter with brightness and contrast adjustments, normalization using ImageNet statistics (mean = [0.485, 0.456, 0.406], std = [0.229, 0.224, 0.225]), and normalization using ImageNet statistics.

### 3.3. Proposed Hybrid Model

This study proposes a hybrid architecture leveraging the complementary strengths of CNNs and transformers. The proposed model consists of a ConvNext-tiny network that extracts local features (see Section 3.3.1), a Swin Transformer that captures global dependencies (see Section 3.3.2), and a fusion module that combines features from both networks. Given an input image x, the models generate two separate 768-dimensional feature embeddings as

(1) $$f_{c}=ConvNext\left(x\right)\;\;\;\;\;f_{s}=SwinTransformer\left(x\right)$$

Here, $f_{c}$ and $f_{s}$ represent the local (CNN-based) and global (Transformer-based) feature descriptors, respectively. This dual-encoder strategy allows the model to capture both fine-grained spatial patterns and long-range dependencies within the malware images.

#### 3.3.1. ConvNext-Tiny

Our CNN-only model utilizes ConvNeXt-Tiny [54], a state-of-the-art CNN architecture incorporating modern design principles from Vision Transformers while maintaining the convolutional structure. ConvNeXt modernizes the ResNet architecture by incorporating the following: larger kernel sizes, fewer activation functions, layer normalization instead of batch normalization, and an inverted bottleneck design.

**Figure 2:**
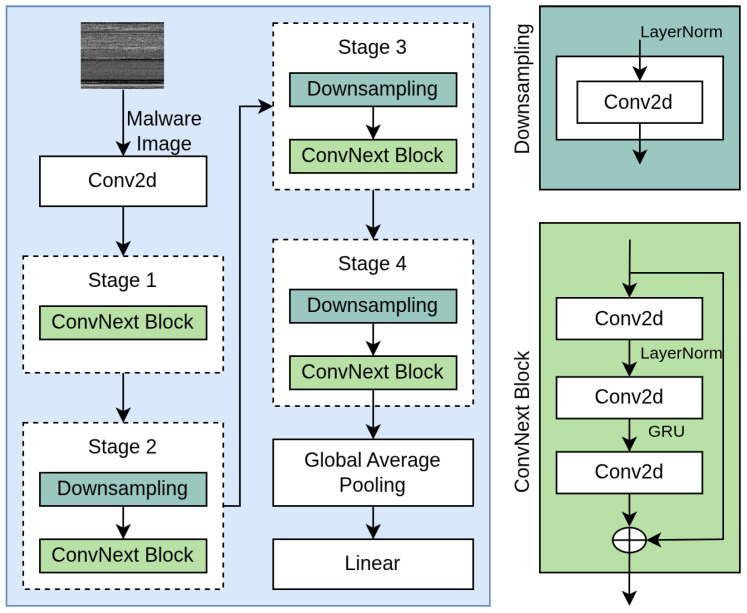
ConvNext architecture.

Figure 2 illustrates the architecture of a ConvNeXt-based model used for malware image classification [55]. The process begins with a 2D convolution applied to the input malware image, followed by four sequential stages. Each stage consists of one or more ConvNeXt blocks, with downsampling applied from Stage 2 onward to reduce spatial dimensions while preserving critical features. The ConvNeXt blocks follow a structured flow: an initial convolution is followed by layer normalization and additional convolutions, with a GRU connection incorporated to help retain important sequence-related information. The output from Stage 4 passes through global average pooling to reduce the feature map to a fixed-size vector, which is then fed into a linear layer for classification. This setup allows the model to learn both low-level and high-level patterns efficiently across the spatial hierarchy, making it well suited for visual malware analysis. We initialized the model with weights pretrained on ImageNet and fine-tuned all layers on malware datasets.

#### 3.3.2. Swin Transformer

**Figure 3:**
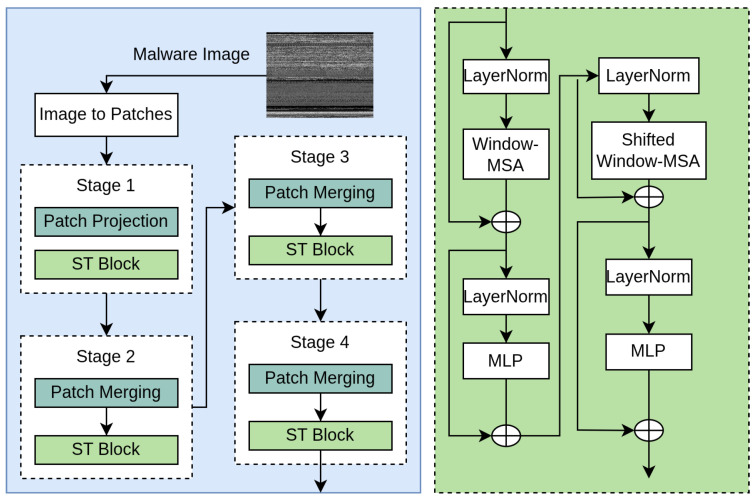
Swin Transformer architecture.

We implemented the Swin Transformer [39] for Transformer-only approach, demonstrating superior performance in various computer vision tasks. Figure 3 presents a Swin Transformer architecture tailored for classifying malware represented as images. The process begins by splitting the malware image into smaller patches, which are then passed through several stages involving patch projection and patch merging. Each stage integrates Swin Transformer blocks, allowing the model to gradually reduce spatial dimensions while capturing increasingly complex patterns. The Swin Transformer block, shown on the right, applies layer normalization, window-based multi-head self-attention, shifted window attention, and multi-layer perceptrons, all linked through skip connections to support effective learning. Key features include hierarchical feature maps similar to CNNs, linear computational complexity concerning image size, and shifted window-based self-attention for cross-window connections.

The Swin-Tiny configuration consists of four stages with increasing feature dimensions and decreasing resolution. As with the CNN model, we initialized the Transformer with ImageNet pretrained weights and fine-tuned all layers on our malware datasets. The fusion module concatenates the feature vectors from both networks (each with 768 dimensions) and processes them through a sequence of fully connected layers with dropout for regularization. Specifically, the fusion module consists of a linear layer that reduces the concatenated 1536-dimensional vector to 512 dimensions, a ReLU activation function, a dropout layer with a rate of 0.4, and a final linear layer that maps to the number of classes (61). Both the CNN and Transformer components are initialized with ImageNet pretrained weights. This hybrid architecture enables the model to simultaneously leverage the local pattern recognition capabilities of CNNs and the global context modeling of Transformers.

#### 3.3.3. Auxiliary Supervision

To enhance gradient flow and stabilize training, auxiliary classification heads are attached to each feature stream. These heads produce class logits ${\hat{y}}_{c}^{aux}$ and ${\hat{y}}_{s}^{aux}$, each of dimension 61, corresponding to the number of malware categories. Cross-entropy losses are computed independently for both streams as $L_{aux}=CE\left({\hat{y}}_{c}^{aux},\;y_{i}\right)+CE\left({\hat{y}}_{s}^{aux},\;y_{i}\right)$. This auxiliary loss encourages both backbones to learn semantically discriminative representations before fusion, facilitating better final predictions.

#### 3.3.4. Feature Fusion and Weighting

The next stage involves concatenating the two feature vectors into a combined representation $f=\left[f_{c}∥f_{s}\right]\in R^{1536}$. This concatenated vector is passed through a gating MLP that outputs two scalar weights $w_{c}$ and $w_{s}$, which are normalized via softmax. These weights represent the learned importance of each feature stream. A weighted feature vector is then constructed as $f_{fusion}=\left[w_{c}\cdot f_{c}∥w_{s}\cdot f_{s}\right]\in R^{1536}$. This adaptive weighting ensures that the model can dynamically prioritize either local (CNN) or global (Transformer) features depending on the input characteristics.

#### 3.3.5. Final Classification

The fused feature vector $f_{f}usion$ is passed through a fusion MLP, which reduces its dimensionality from 1536 to 512 using a linear layer followed by ReLU activation and dropout. This intermediate representation is then passed through a final fully connected layer mapping $R^{512}\to R^{61}$, producing logits for each class. A softmax function is applied to these logits to compute the final prediction probabilities $\hat{y}$ for classification.

#### 3.3.6. Knowledge Distillation

To further improve generalization and transfer knowledge from high-performing teacher models, the architecture incorporates knowledge distillation. Pretrained ConvNeXt and Swin models act as frozen teachers, generating soft targets ${\hat{y}}_{teacher}$. The student’s predictions are compared to these soft targets using the Kullback–Leibler divergence loss defined as $L_{distill}=KL\left(\hat{y},\;{\hat{y}}_{teacher}\right)$. Distillation encourages the student model to mimic the output distributions of the teachers, promoting smoother decision boundaries and better generalization.

#### 3.3.7. Total Loss and Backpropagation

The total loss function is a weighted sum of the main classification loss, auxiliary supervision loss, and distillation loss defined as $L=CE\left(\hat{y},\;y_{i}\right)+\lambda_{1}L_{aux}+\lambda_{2}L_{distill}$. Here, $\lambda_{1}$ and $\lambda_{2}$ control the contributions of auxiliary and distillation components, respectively. The network parameters are updated through backpropagation using gradient-based optimization (AdamW), with mixed-precision training and gradient clipping applied to stabilize convergence.

#### 3.3.8. Validation

After each training epoch, the model is assessed on a distinct validation set covering all three datasets. Validation accuracy and loss are monitored, and the best-performing model is preserved. Early stopping is triggered if the validation accuracy fails to enhance for a specified number of epochs, preventing overfitting and saving computational resources.

### 3.4. Experimental Setup

This study was conducted using the Google Colab Pro platform, leveraging the NVIDIA A100 GPU environment. The computational setup included 83.5 GB of system RAM and 40.0 GB of dedicated GPU memory, enabling efficient training and evaluation of deep learning models. This high-performance configuration facilitated rapid experimentation with complex architectures and large-scale malware image datasets. We used Python version 3.11.13 along with the following packages: PyTorch 2.6.0+cu124, Matplotlib 3.10.0, NVIDIA CUDA NVCC (cu12) 12.5.82, NVIDIA cuDNN (cu12) 9.3.0.75, OpenCV-Python 4.12.0.88, Pandas 2.2.2, numpy==2.0.2, and Seaborn 0.13.2. We trained the proposed model using the following configurations: batch size = 128, AdamW as optimizer with a weight decay of 1 × 10^−3^ and learning rate of 5 × 10^−5^, epochs = 20, mixed precision training using PyTorch’s automatic mixed precision, and gradient clipping with a maximum norm of 1.0. For the hybrid model, we also explored a two-phase training approach: initial training of all components and fine-tuning only the fusion layers while keeping the CNN and Transformer components frozen. This approach allowed the model to learn effective representations from both architectural paradigms and then focus on optimizing the fusion strategy. We used a lower learning rate (1 × 10^−6^) and a cosine annealing schedule during fine-tuning to gradually reduce the learning rate over 10 epochs.

### 3.5. Model Evaluation

In anomaly detection, relying solely on accuracy can be misleading, especially when normal events heavily outnumber anomalies. That is why precision, recall, and F1-score are also significant. Precision shows how many flagged anomalies were correct, while recall reflects how many real anomalies the model was able to catch. The F1-score gives a balanced view between the two. For instance, if a model correctly identifies 15 anomalies out of 25 flagged, misses 5 real ones, and incorrectly marks 10 normal cases, the accuracy might still seem high at 98.5%. However, the precision would be 60%, recall 75%, and the F1-score around 66.7%, giving a clearer picture of its actual effectiveness. Equations (2), (3), (4) and (5) show calculation of the accuracy, precision, recall, and F1-score, respectively. True Positive (TP) is when an actual anomaly is correctly detected, False Positive (FP) is when normal data are mistakenly flagged as anomalies, True Negative (TN) is correctly identifying normal data, and False Negative (FN) is when the model fails to catch a real anomaly.

(2) $$Accuracy=\frac{TP+TN}{TP+FP+TN+FN}$$

(3) $$Precision=\frac{TP}{TP+FP}$$

(4) $$Recall=\frac{TP}{TP+FN}$$

(5) $$F1-score=\frac{2\times Precision}{Precision+Recall}$$

## 4. Result

**Figure 4:**
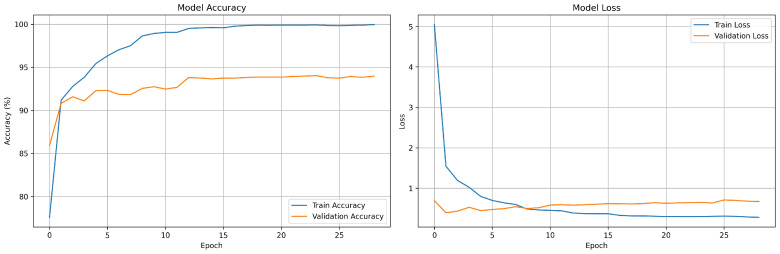
Training and validation accuracy–loss per epoch.

Figure 4 illustrates the training and validation performance of the model across 28 epochs. The training accuracy (left) steadily increased and approaches 100%, while the validation accuracy rose quickly in the early epochs before stabilizing around 94%. The validation loss leveled off and exhibited slight fluctuations after the initial drop. These trends indicate that while the model learns the training data very well, its ability to generalize to unseen data is reasonable.

Table 3 compares the classification performance of three different deep learning approaches—CNN-only, Transformer-only, and a hybrid of both—in the MallImg, MaleVis, and VirusMNIST datasets. The training times for the CNN-only, Transformer-only, and hybrid models were 2:14, 2:03, and 2:43 hours:minutes, respectively. We list the testing times in seconds for these three models across three datasets. The first two models had less training time due to early stopping, which halted training after 17 epochs. In contrast, the hybrid model continued training for 29 epochs before early stopping was triggered, resulting in its longer total training duration. Additionally, the results show that the hybrid model consistently outperformed individual architectures, achieving the highest average accuracy of 94.04%. Although the CNN-only model performed well on MallImg and MaleVis, and the Transformer-only model showed strength in VirusMNIST, neither surpassed the hybrid approach, which balanced high accuracy with strong precision, recall, and F1-scores across all datasets. These results highlight the benefit of combining CNNs’ local feature extraction with Transformers’ global context understanding to improve malware image classification.

Table 4 presents the evaluation metrics—precision, recall, and F1-score—for various malware classes across three datasets: MallImg, MaleVis, and VirusMNIST. The results show that most classes achieved perfect or near-perfect scores on the MallImg dataset, indicating strong model performance. For MaleVis, while the majority of classes also scored highly, a few, such as Agent-fyi and Adposhel, had slightly lower values, reflecting some variation in classification accuracy. VirusMNIST, which includes more diverse and possibly harder-to-distinguish samples, showed greater variation across classes, with some like class 0 and class 5 scoring lower, especially in recall and F1-score. Overall, the metrics suggest that the model performed consistently well, with minor challenges in detecting certain classes, particularly in the VirusMNIST dataset.

**Figure 5:**
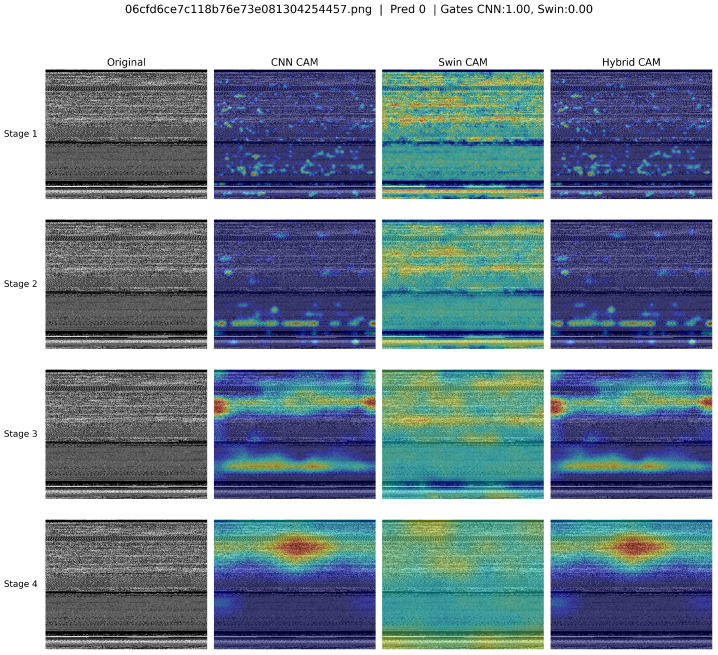
Grad-CAM visualization comparison of ConvNeXt (CNN), Swin Transformer, and hybrid models across four different stages, highlighting areas of attention for classification.

Figure 5 depicts the Grad-CAM visualizations, which clearly illustrate how the CNN (ConvNeXt), Swin Transformer, and the hybrid model responded differently across four distinct stages. At stage 1, the CNN highlighted scattered areas, reflecting selective attention to localized features, whereas the Swin Transformer showed more widespread attention across the input. The hybrid model effectively merged these two behaviors, capturing both localized and broader areas. In stages 2 and 3, the CNN CAM began to intensify and cluster more around specific central regions, while the Swin CAM maintained consistent, broader attention coverage. At stage 4, all models strongly highlighted a prominent central area, suggesting a clear consensus on critical regions for classification. Overall, the hybrid model successfully integrates the strengths of ConvNeXt and Swin Transformer, providing both detailed and global interpretability and indicating robust and complementary feature extraction.

**Figure 6:**
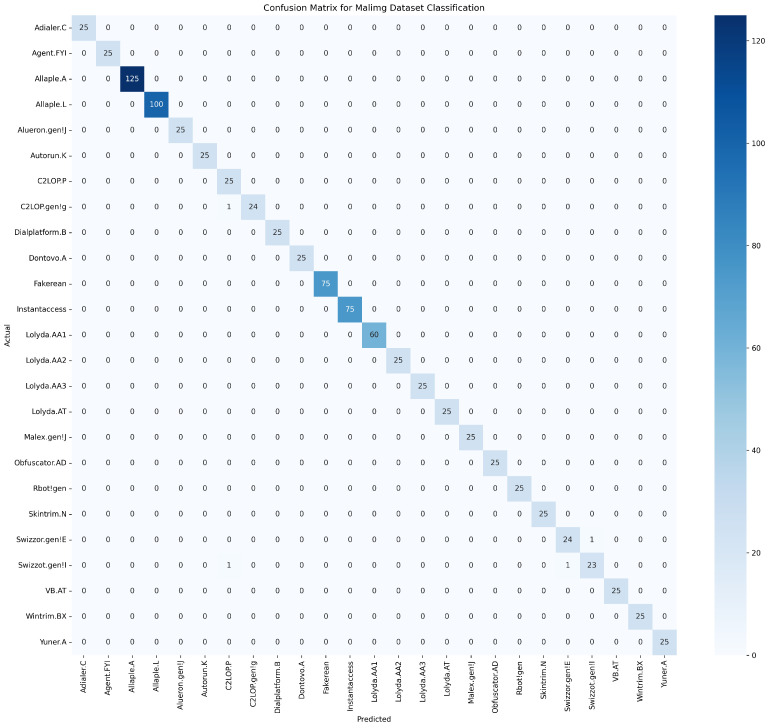
Confusion matrix for Malimg dataset classification.

Figure 6 illustrates the confusion matrix for the Malimg dataset classification. The proposed model demonstrated high accuracy, reflected by the pronounced diagonal entries indicating correct predictions for each malware family. However, minor misclassifications appeared, notably between the Swizzor.gen!E and Swizzor.gen!I families, suggesting subtle feature overlaps that might require refined feature extraction methods or improved model differentiation to address these nuances. Overall, the matrix suggests strong classification efficacy, with potential areas for further fine-tuning to enhance discriminative capabilities between closely related malware classes.

**Figure 7:**
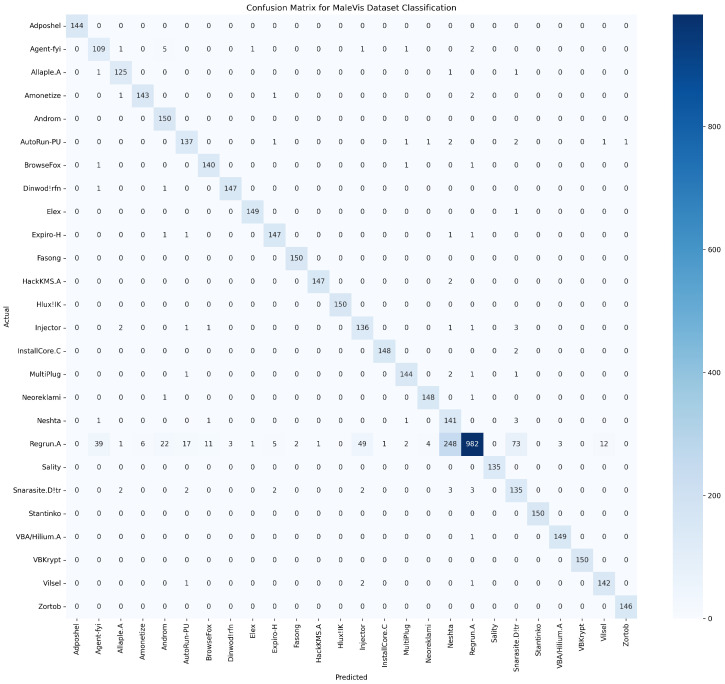
Confusion matrix for MaleVis dataset classification.

Figure 7 demonstrates the confusion matrix of the proposed model evaluated in the MaleVis dataset. Most malware families had outstanding classification accuracy, as evidenced by pronounced diagonal elements, notably the Regrun—a category which displays a high count of correct identifications. Nonetheless, notable misclassifications emerged for Regrun.A, which was frequently confused with multiple other malware families, indicating potential ambiguities or overlapping behavioral characteristics within these classes.

**Figure 8:**
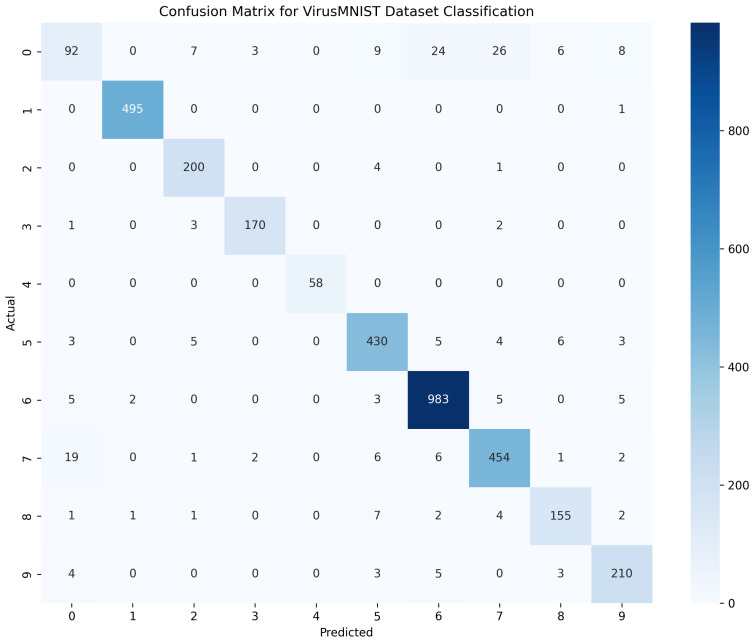
Confusion matrix for VirusMNIST dataset classification.

Figure 8 shows the confusion matrix for the VirusMNIST dataset classification, showing generally strong model performance, with clear diagonal dominance that indicates accurate predictions in most families. Class ‘6’ notably achieved high accuracy, as evidenced by the diagonal entry. However, the model struggled somewhat with class ‘0’, misclassifying several instances into classes ‘5’, ‘6’, and ‘7’, highlighting feature ambiguities or insufficient distinction in these cases.

Table 5 presents a comparative overview of recent studies related to malware and malicious content detection using various deep learning methodologies. The progression of techniques from CNN-based models in 2018 to more sophisticated architectures such as ViTs and hybrid models in 2024 reflects a clear trend toward improving accuracy and generalization. While early methods, like CNN and ensemble learning, achieved respectable performance (e.g., 92.11% on MallImg), more recent approaches such as ResNet-152, LeViT-MC, and Butterfly ViT have demonstrated significantly higher accuracies, with some exceeding 99%. However, a recurring limitation across these methods is the lack of model generalization and absence of explainability tools like GRAD-CAM. In contrast, the current study stands out for its attempt to build a generalized hybrid model that performs well across diverse datasets (MallImg, MaleVis, and VirusMNIST), showing competitive accuracies (e.g., 99.25% on MallImg). The main trade-off noted is the need for further optimization to enable real-time deployment, highlighting a practical challenge often overlooked in previous research.

In addition to the three benchmark datasets, we have extended our validation to two more datasets—Maldeb and Dumpware-10—to strengthen the empirical foundation of our work. These datasets introduce both binary and multi-class classification challenges, encompassing a broader range of malware families and structural diversity. The proposed hybrid model achieved competitive accuracy results on both the Maldeb and Dumpware-10 datasets. This expanded evaluation reinforces the model’s generalization ability and practical applicability across varied threat landscapes.

Table 6 presents the validation results of the proposed hybrid model on two additional malware image datasets, Maldeb and Dumpware-10, extending the evaluation beyond the original three benchmarks. The Maldeb dataset consists of binary classes (benign and malicious), while Dumpware-10 comprises 10 malware families and one benign class. All images were uniformly resized to 224 × 224 pixels to align with the model’s input requirements. The model achieved a 98% accuracy result on Maldeb, improving upon the previously reported best performance of 96.2% [35]. On Dumpware-10, it attained a 97% accuracy result, which—although slightly lower than the 98.19% reported in prior work [36]—remains competitive. These improvements confirm the effectiveness of the proposed hybrid fusion approach. Moreover, the results span both binary (Maldeb, 2 classes) and multi-class (Dumpware-10, 11 classes) classification settings, affirming the model’s generalization ability.

Table 7 presents class-wise precision, recall, and F1-score results for the Maldeb and Dumpware-10 datasets. The model achieved balanced results on Maldeb, with both benign and malicious classes scoring an F1 of 0.98. For Dumpware-10, most classes exceeded 0.95 in F1-score, with top performance on Adposhel, InstallCore, and VBA. Slightly lower scores on AutoRun and Dinwod suggest room for improvement in those categories.

## 5. Real-World Deployment Scenario

In a real-world cybersecurity infrastructure—such as within an enterprise security operations center (SOC) or at a network gateway level—the proposed hybrid malware classification model can function as a second-tier detection engine to enhance threat visibility and response speed.

Consider a scenario where files or executables enter the network via email attachments, USB drives, or downloads. As part of the organization’s security pipeline, each file is immediately passed through a lightweight feature extraction module that converts the binary content into an image representation—using techniques such as byte-to-pixel mapping or grayscale rendering. This transformation is done on the fly and is compatible with the preprocessing requirements of the model (resizing, normalization, etc.). Converting malware binaries into images does introduce some preprocessing overhead, but the trade-off is justified. This step allows us to apply powerful vision-based models that can capture structural and distributional patterns in the binary data, which traditional methods might miss. The conversion is a one-time operation, and it was efficiently handled in our setup using GPU acceleration.

Once the malware image is generated, it is fed into the deployed hybrid model. The model performs real-time inference using GPU-accelerated computation—extracting features in parallel from both the ConvNeXt and Swin Transformer backbones, fusing them using dynamic gating, and producing a classification output within milliseconds. This classification result indicates the most probable malware family or flags the file as benign.

Security analysts or automated response systems can then take action based on the prediction as follows:

- High-confidence malware (e.g., 99% confidence in a known ransomware family) can trigger automatic quarantine or blocking.
- Uncertain predictions can be passed to human analysts for manual triage.
- Explainability is provided through Grad-CAM visualizations, helping analysts understand which parts of the malware structure influenced the model’s decision, increasing transparency and trust.

This setup also allows for continuous learning—new malware variants can be collected, labeled, and used to fine-tune the model periodically. Additionally, the auxiliary heads and knowledge distillation framework enable efficient training and deployment even in environments with constrained resources, such as IoT gateways or mobile endpoint security agents.

### 5.1. Trade-Offs in Visual Malware Classification

Converting malware binaries to images enables powerful vision models (e.g., CNNs, ViTs) that can capture both local and global patterns, improving detection accuracy and allowing interpretability through techniques like Grad-CAM. This is especially useful in environments needing transparency, such as SOCs. However, this approach demands high computational resources and introduces preprocessing overhead, making it less suitable for real-time or edge deployments. Visual methods may also miss important sequential or behavioral information and require large, diverse datasets to handle obfuscated malware effectively. Overall, byte-to-image classification works best in offline or cloud settings where accuracy and explainability outweigh latency and resource constraints. For lightweight or real-time needs, alternative or hybrid approaches may be necessary.

### 5.2. Limitations of the Study

While the proposed hybrid model demonstrates strong performance, several limitations can be specified. One notable constraint is the preprocessing overhead introduced by converting raw binaries into image representations. Although the conversion process is manageable in GPU-equipped environments like SOCs, its computational needs create challenges for use in low-power edge devices. This limitation may restrict the model’s applicability in environments where minimal latency and efficiency are critical. Furthermore, while Grad-CAM provides valuable visual alerts by indicating which regions influence predictions, this type of interpretability remains confined to the image domain. In practical cybersecurity settings, analysts often require deeper insights—such as logical associations or behavior-level explanations—to fully understand and trust automated detection outputs.

## 6. Conclusions and Future Work

In this study, the Transformer backbone was selected for its global context modeling capability, which complements the local spatial sensitivity of CNNs. The hybrid architecture was designed to exploit this complementarity—ConvNeXt captures fine-grained local features, while Swin Transformer provides broader contextual understanding. We conducted a detailed comparison of three models, CNN-only, Transformer-only, and a hybrid combining both, to classify malware based on visual features. Although the standalone Transformer model was less accurate than ConvNeXt in isolation, our adaptive fusion mechanism successfully integrated their respective strengths, leading to an improved hybrid performance of 94.01%. Additionally, we validated our model on the Maldeb and Dumpware-10 datasets, achieving promising accuracy scores of 98% and 97%, respectively.

A key aspect of our approach was to use Grad-CAM to visualize which parts of the images the models focused on. Interestingly, we found that Transformer models showed little activation when processing malware visuals, suggesting that they might not be as effective in identifying critical features in this domain. In contrast, CNNs were more attentive to local visual patterns, while Transformers captured broader spatial relationships. These behaviors complement each other in the hybrid architecture, which benefits from both local detail and global context. A layer-wise examination of both models confirmed a step-wise learning process, where simpler patterns are captured early and more abstract features emerge in deeper layers.

Overall, our findings highlight the advantages of hybrid CNN–Transformer architectures, especially when paired with visualization techniques that improve model interpretability. As for the next steps, we plan to refine how CNNs and Transformers are integrated to better exploit their strengths. Since Transformer-only models appeared less effective with malware images, their design or training process adjustments may help improve their performance in this niche. We are also interested in making these models lighter and more efficient—essential factors for real-world cybersecurity systems that may operate under hardware constraints. Approaches like pruning, quantization, or knowledge distillation could play a role here. Although the current model may not be optimal for resource-constrained edge environments, it is well suited for deployment within enterprise SOCs or cloud-based infrastructures where GPU acceleration is available. In such settings, it can operate in real time as a second-tier detection engine, enhancing decision accuracy and providing interpretability through Grad-CAM visualizations—key for analyst trust and effective incident response. Finally, we intend to expand our testing to larger and more varied malware datasets to understand better how these models hold up in different threat environments.

## Figures and Tables

**Table 1:** Summary of related malware classification studies.

Article	Methods	Dataset and Result	Objective
[27], 2025	Kolmogorov–Arnold Network and GAN	Malimg: 99.36%, MaleVis: 95.44%, VirusMNIST: 92.12%	Lightweight ensemble for malware detection
[17], 2025	Layered feature extractor + spatial-CNN	Malimg: 99.87%, BIG2015: 99.81%, MaleVis: 99.22%	Propose efficient layered architecture
[18], 2025	ResNet50+ VGG16 with knowledge distillation	Malimg: 99.50%, BIG2015: 97.52%	Compress deep models with fused layers
[42], 2024	Self-supervised Swin Transformer	BIG2015: 97.85%, Malimg: 98.28%	Lightweight framework for malware classification
[45], 2024	Dual ViT with split attention	Ransomware dataset: 99.99%	Detect ransomware with Vision Transformer
[19], 2024	DenseNet201-KNN	Malimg: 96%	Benchmark pretrained models on malware
[43], 2024	Swin Transformer with deformable attention	Malimg: 99.35%	Improve classification via attention mechanism
[13], 2024	CNN and ensemble learning	MalImg: 99.36%, real-world malware dataset: 92.11%	Transfer learning and ensemble learning for malware classification
[41], 2024	ResNet-152 and ViT	MalNet: 99.62%	Transformer-based CNN model for malware classification
[34], 2023	InceptionV3	BIG15: 98.76%	Compare ML and TL for malware detection
[33], 2023	CNN and feature fusion set	Malware dataset: 97% accuracy	Feature fusion for malware classification
[15], 2023	CNN with attention layer	Malimg: 99%	Enhance robustness in detection
[47], 2023	Lightweight CNN, ViT, CCT, EANet; LIME	Malimg: 99.44% (CNN)	Compare CNN vs Transformer on malware images
[25], 2023	CNN, MobileNetV2, ResNet-50	Malimg: 100%	Evaluate pretrained models
[20], 2023	CNN, DNN	Malimg: 98.14%, BIG2015: 98.95%	Broad DL-based malware analysis
[40], 2023	Butterfly ViT	MalImg: 99.32%, Microsoft Big: 99.49%, PE imports: 99.99%	Global–local attention-based malware classification
[44], 2022	SFTA-GDA and NB	MaleVis: 98%	Binary and multi-class classification
[14], 2018	CNN	MalImg: 98.52%, Microsoft: 99.97%	Analysis CNN performance for malware

**Table 2:** Malware families and threat types.

Threat	Families
Dialer	Adialer.C, Dialplatform.B, Instantaccess
Backdoor	Agent.FYI, Rbot!gen
Worm	Allaple.A, Allaple.L, Autorun.K, Autorun.PU, VB.AT, Yuner.A, Fasong
Trojan	Alueron.gen!J, C2LOP.P, C2LOP.gen!g, Malex.gen!J, Skintrim.N, Androm, Dinwod, Dinwod!rfn, Expiro-H, Injector, Regrun.A, Snarasite.D!tr, Stantinko, VBKrypt, Vilsel
TDownloader	Dontovo.A, Obfuscator.AD, Swizzor.gen!E, Swizzot.gen!I, Wintrim.BX, Neshta
Rogue	Fakerean
PWS	Lolyda.AA1, Lolyda.AA2, Lolyda.AA3, Lolyda.AT
Adware	Adposhel, MultiPlug
PUA	Amonetize, BrowseFox, Elex
Behavior	InstallCore.C, Neoreklami
TDropper	Sality
Virus	VBA/Hilium.A

**Table 3:** Performance comparison of CNN-only, Transformer-only, and proposed hybrid models across three benchmark malware datasets using training and testing time and evaluation metrics.

Method	Dataset	Training Time (H:M)	Testing Time (s)	Acc	Pr	Re	F1-Score	Avg Acc
CNN-Only	MalImg	2:14	3.90	96.36	92.58	95.56	93.23	92.45
	MaleVis		19.81	88.47	90.70	96.40	92.56	
	VirusMNIST		7.52	93.26	91.71	91.10	91.26	
Transformer-Only	MalImg	2:03	4.76	95.94	91.98	93.92	92.60	90.44
	MaleVis		20.00	88.35	90.31	95.79	92.15	
	VirusMNIST		10.51	92.05	89.00	90.23	89.46	
Hybrid	MalImg	2:43	4.29	99.57	99.38	99.36	99.36	94.04
	MaleVis		20.15	88.65	90.74	96.49	92.69	
	VirusMNIST		8.12	93.90	92.44	91.42	91.74	

**Table 4:** Evaluation metrics for individual malware classes across MallImg, MaleVis, and VirusMNIST datasets.

MalImg				MaleVis				VirusMNIST			
**Class**	**Pr**	**Re**	**F1**	**Class**	**Pr**	**Re**	**F1**	**Class**	**Pr**	**Re**	**F1**
Adialer.C	1.00	1.00	1.00	Adposhel	1.00	1.00	1.00	0	0.74	0.53	0.61
Agent.FYI	1.00	1.00	1.00	Agent-fyi	0.71	0.91	0.80	1	0.99	1.00	1.00
Allaple.A	1.00	1.00	1.00	Allaple.A	0.95	0.98	0.96	2	0.92	0.98	0.95
Allaple.L	1.00	1.00	1.00	Amonetize	0.95	98	0.97	3	0.97	0.97	0.97
Alueron.gen!J	1.00	1.00	1.00	Androm	0.83	1.00	0.91	4	1.00	1.00	1.00
Autorun.K	0.92	1.00	0.96	AutoRun-PU	0.86	0.94	0.90	5	0.93	0.94	0.94
C2LOP.P	1.00	0.96	0.98	BrowseFox	0.92	0.98	0.96	6	0.96	0.98	0.97
C2LOP.gen!g	1.00	1.00	1.00	Dinwod!rfn	0.98	0.99	0.98	7	0.92	0.92	0.92
Dialplatform.B	1.00	1.00	1.00	Elex	0.99	0.99	0.99	8	0.91	0.90	0.90
Dontovo.A	1.00	1.00	1.00	Expiro-H	0.94	0.97	0.96	9	0.91	0.93	0.92
Fakerean	1.00	1.00	1.00	Fasong	0.99	1.00	0.99				
Instantaccess	1.00	1.00	1.00	HackKMS.A	0.99	0.99	0.99				
Lolyda.AA1	1.00	1.00	1.00	Hlux!IK	1.00	1.00	1.00				
Lolyda.AA2	1.00	1.00	1.00	Injector	0.72	0.94	0.81				
Lolyda.AA3	1.00	1.00	1.00	InstallCore.C	0.99	0.99	0.99				
Lolyda.AT	1.00	1.00	1.00	MultiPlug	0.96	0.97	0.96				
Malex.gen!J	1.00	1.00	1.00	Neoreklami	0.97	0.99	0.98				
Obfuscator.AD	1.00	1.00	1.00	Neshta	0.35	0.96	0.51				
Rbot!gen	1.00	1.00	1.00	Other	0.99	0.66	0.79				
Skintrim.N	1.00	1.00	1.00	Regrun.A	1.00	1.00	1.00				
Swizzor.gen!E	0.96	0.96	0.96	Sality	0.61	0.91	0.73				
Swizzor.gen!I	0.96	0.92	0.94	Snarasite.D!t	1.00	1.00	1.00				
VB.AT	1.00	1.00	1.00	Stantink	0.98	0.99	0.99				
Wintrim.BX,	1.00	1.00	1.00	VBA	1.00	1.00	1.00				
Yuner.A.	1.00	1.00	1.00	VBKrypt	0.92	0.97	0.94				
				Vilsel	0.99	1.00	0.99				

**Table 5:** Comparative analysis of recent methodologies for malware classification, highlighting accuracy, strengths, and limitations.

Year	Methodology	Dataset & Accuracy	GRAD-CAM
2024 [13]	CNN and ensemble learning	MalImg: 99.36%, real-world malware dataset: 92.11%	No
2024 [41]	ResNet-152 and ViT	MalNet: 99.62%	No
2024 [44]	SFTA-GDA and NB	MaleVis: 98%	No
2023 [40]	Butterfly ViT	MalImg: 99.32%, Microsoft Big: 99.49%, PE imports: 99.99%	No
2018 [14]	CNN	MalImg: 98.52%, Microsoft: 99.97%	No
This study	Hybrid	MalImg: 99.57%, MaleVis: 88.65%, VirusMNST: 93.90%	Yes

**Table 6:** Performance of the proposed hybrid model evaluated on Maldeb and Dumpware-10 datasets.

Dataset	Accuracy	Precision	Recall	F1-Score	Classes
Maldeb	0.98	0.98	0.98	0.98	2
Dumpware-10	0.97	0.97	0.95	0.96	11

**Table 7:** Evaluation metrics for individual malware classes across Maldeb and Dupmware-10 datasets.

Maldeb				Dumpware-10			
**Class**	**Pr**	**Re**	**F1**	**Class**	**Pr**	**Re**	**F1**
Benign	0.99	0.98	0.98	Adposhel	1.00	1.00	1.00
Malicious	0.98	0.99	0.98	Allaple	1.00	0.98	0.99
				Amonetize	0.99	0.95	0.97
				AutoRun	0.89	0.82	0.85
				BrowseFox	0.95	1.00	0.97
				Dinwod	0.96	0.83	0.89
				InstallCore	0.99	0.99	0.99
				MultiPlug	0.94	0.98	0.96
				Other	0.92	0.97	0.94
				VBA	0.99	1.00	1.00
				Vilsel	1.00	0.99	0.99

## Data Availability

The data presented in this study were derived from publicly available sources and can be accessed through the following repositories: Malimg (https://figshare.com/articles/dataset/MalImg_dataset_zip/24189882?file=42443904, accessed on 15 June 2025), Virus-MNIST (https://www.kaggle.com/datasets/datamunge/virusmnist, accessed on 15 June 2025), Malevis (https://www.kaggle.com/datasets/sohamkumar1703/malevis-dataset, accessed on 15 June 2025), Maldeb (https://www.kaggle.com/datasets/saquib7hussain/maldeb-dataset, accessed on 15 June 2025), and Dumpware-10 (https://www.kaggle.com/datasets/nenniramziwassim/dumpware-10, accessed on 15 June 2025).
